# The dynamic world of RNA: beyond translation to subcellular localization and function

**DOI:** 10.3389/fgene.2024.1373899

**Published:** 2024-03-12

**Authors:** Annika Reisbitzer, Sybille Krauß

**Affiliations:** University of Siegen, Institute of Biology, Human Biology / Neurobiology, Siegen, Germany

**Keywords:** RNA-localization, RNA-transport, alternative polyadenylation, neurodegenerative diseases, zipcodes

## 1 Introduction

RNA is a versatile and dynamic molecule that plays an important role in many aspects of cell biology and physiology. Its diverse functions reach from acting as a template for protein biosynthesis to a variety of regulatory, structural, and catalytic activities in the cell. Primary transcripts and processed RNAs are not randomly distributed in the cell but are found in specific subcellular compartments. This is particularly important in neurons, a highly polarized cell type. Several mechanisms are known to regulate RNA localization and transport, including passive diffusion, phase separation into RNA granules, and active transport. All these processes contribute to cellular RNA dynamics. Interestingly, these transport mechanisms are influenced by different regulatory elements of the RNA molecule itself, e.g., by alternative polyadenylation, or by the association with RNA-binding proteins (RBPs). Deregulation of these mechanisms is connected to pathogenic mechanisms that lead, for example, to neurodegenerative diseases.

## 2 Passive diffusion and phase separation

The process of passive diffusion, which is driven by random molecular motion, enables the distribution of RNA molecules without the need for energy-intensive transport systems or specific guidance elements. For example, RNA diffusion has been observed in cytoplasm during the embryogenesis of *Drosophila* (Forrest and Gavis, 2003; Engel, Arora, Goering, Lo and Taliaferro, 2020). Diffusion also allows the movement of RNA inside the nucleus of mammalian cells (osteosarcoma cell line U2OS) (Shav-Tal et al., 2004). Importantly, diffusion seems to act in concert with other transport mechanisms such as local entrapment mechanisms and active transport to generate asymmetric RNA distribution in cells (Forrest and Gavis, 2003). For example, recent experiments on the oogenesis in *Xenopus* suggest that RNA transcripts localize through diffusion and association with the ER to a structure called the mitochondrial cloud (Chang et al., 2004; Palacios, 2007).

**FIGURE 1:**
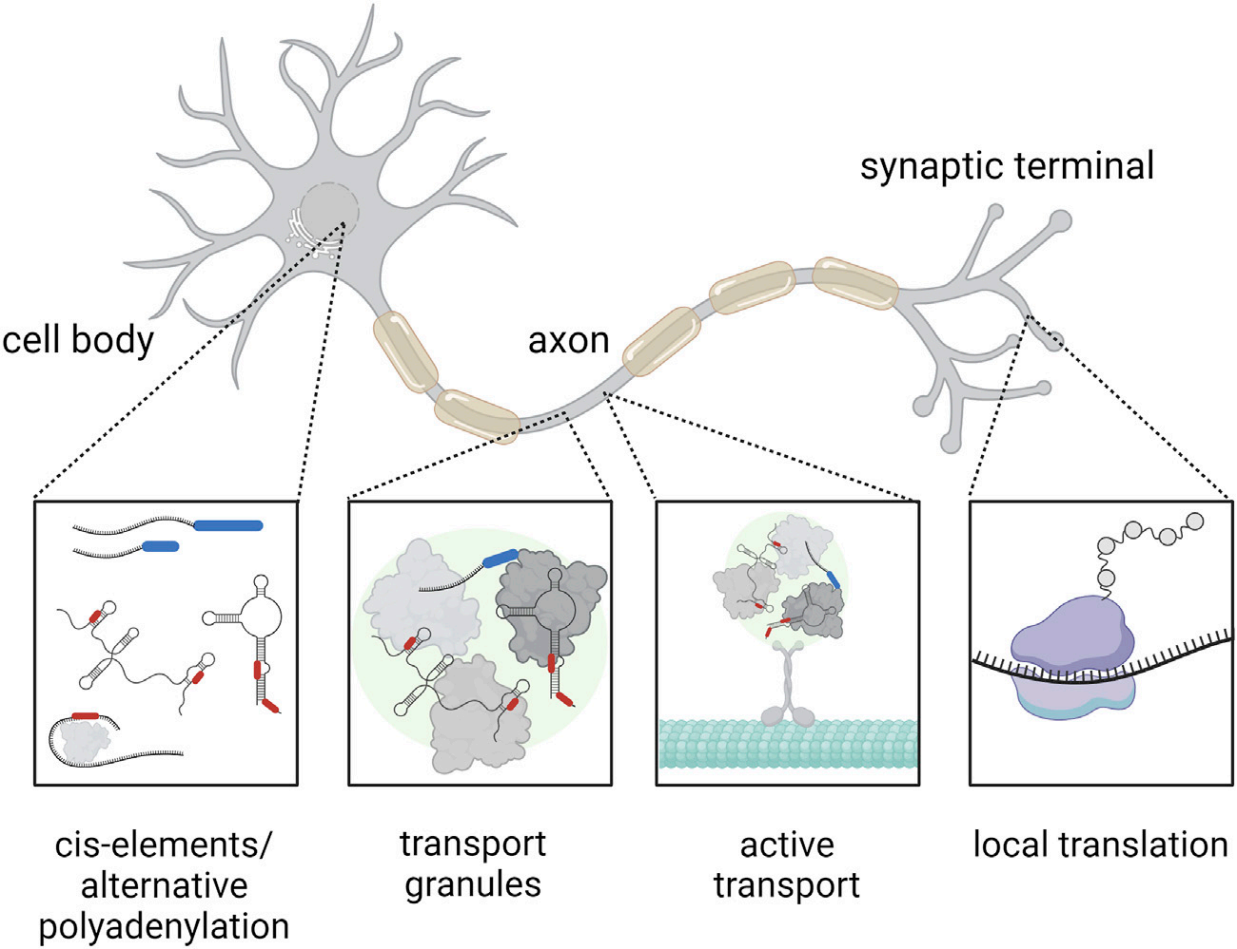
Mechanism of RNA transport within a highly polarized neuron. The RNA (black) contains cis-elements (red) at the UTRs and may feature alternative polyadenylation at the 3′UTR (blue), which represent binding sites for RNA-binding proteins. These RNA-binding proteins (gray) can affect the subcellular localization of RNA, for example, by assembly of RNA transport granules (green), that can be actively transported across the cytoskeleton (cyan). Finally, the RNA can be translated locally at the synapse (the ribosome is indicated in purple). Created with BioRender.com.

In addition to passive diffusion, RNA localization is also regulated by phase separation. Liquid-liquid phase separation drives formation of membrane-less RNA granules, such as stress granules, p-bodies (processing bodies), and RNA transport granules (Figure 1). These granules influence difference aspects of RNA metabolism: p-bodies are the sites for regulation of RNA stability and degradation, RNA transport granules are responsible for RNA transport and localized translation, and stress granules promote translational block during cell stress (reviewed in (Tian, Curnutte and Trcek, 2020)). Thus, liquid-liquid phase separation represents one mechanism by which specific RNAs are directed to specific cellular compartments, allowing precise spatio-temporal regulation of gene expression (reviewed in (Alberti, Gladfelter and Mittag, 2019)).

Although liquid-liquid phase separation guides RNAs to a certain type of RNA granule, this movement is not static. Different types of granules do interact and exchange content, for example, stress granules and p-bodies interact with each other (Kedersha et al., 2005). Single-molecule imaging reveals that direct movement of mRNA from stress granules to p-bodies is possible, but at a low frequency (Wilbertz et al., 2019). In addition, recent evidence shows that although most mRNAs that are recruited to stress granules are translationally silenced, some mRNAs localized to stress granules can undergo translation (Mateju et al., 2020). This indicates that RNA localization and translational regulation in context of RNA granules is very dynamic and has not yet been fully resolved.

## 3 Active RNA transport

Active, directed RNA transport takes place in a variety of organisms and cell types including yeast, *Drosophila* embryo, *Xenopus* oocytes, fibroblasts, and oligodendrocytes (Forrest and Gavis, 2003; Zaessinger, Busseau and Simonelig, 2006; Tadros et al., 2007). Especially in highly specialized and elongated cells such as neurons, the active transport of RNA plays a crucial role. Here both, the microtubule and actin cytoskeleton, serve as transport tracks (Figure 1). Motor proteins such as dyneins, kinesins, and myosins transport RNA-protein complexes along these structures (reviewed in (Engel et al., 2020)). For example, in neurons, transport of mRNAs including *beta-actin* mRNA occurs predominantly along microtubules to axons and dendrites, a process that is critical for synaptic function and plasticity (Eliscovich, Buxbaum, Katz and Singer, 2013). Transport of RNA-protein complexes allows local protein synthesis in cell compartments far away from the cell body, like the synapse. Recent reports show that local protein synthesis takes place at neuronal pre- and postsynaptic compartments (Hafner, Donlin-Asp, Leitch, Herzog and Schuman, 2019). Although a growing number of proteins and transcripts that play a role in translation regulation at the synapse are continuously identified further studies are required to understand the exact molecular mechanisms.

## 4 RNA regulatory elements: cis-elements and alternative polyadenylation

Cis-elements, also known as zip codes, are also part of the localization machinery of RNA in cells. These sequences are located primarily in the untranslated regions (UTRs) of mRNAs and are recognized by RBPs, which facilitate the active transport of RNA to specific cellular locations (Figure 1). For example, specific zip codes in the 3′UTR of *beta-actin* mRNA control its localization in the peripheral cytoplasm (Kislauskis, Zhu and Singer, 1994). Importantly, different motifs in the UTRs may have different functions with respect to regulating RNA localization: while one UTR can direct the RNA to a general cellular region like the neurites, the other UTR could support the precise subregional localization, e.g., the synapse (Meer et al., 2012; Merianda, Gomes, Yoo, Vuppalanchi and Twiss, 2013). Moreover, processing of the UTRs can contribute to the regulation of an mRNA subcellular localization. Several studies indicate that pre-mRNA processing in the nucleus allows recruitment of RBPs that promote transport to the cytoplasm (reviewed in (Martin and Ephrussi, 2009)). In neurons 3′UTR cleavage may direct transcripts to axons (Andreassi et al., 2021). These findings highlight the importance of zip codes for intracellular RNA targeting, a process crucial for cell polarization and differentiation. However, we are just beginning to understand the function of zip codes and only a fraction of these cis-elements for RNA localization have been discovered to date.

Another type of regulatory elements that affects RNA localization is alternative polyadenylation, which leads to the generation of mRNA isoforms with different 3′UTRs. This allows cells to fine-tune gene expression spatially and temporarily, which is essential for processes such as development and differentiation. Alternative polyadenylation also contributes to neural-specific expression patterns by generating transcripts with different 3′UTRs, which preferentially localize to different neuronal compartments (reviewed in (Arora et al., 2022)). One example is the *brain-derived neurotrophic factor* (*BDNF*) RNA. The two isoforms with different 3′UTRs are differently localized within neurons: the *BDNF* mRNA with short 3′UTR is found in the soma and the *BDNF* mRNA with long 3′UTR is transported to dendrites, contributing to synaptic plasticity (An et al., 2008). Moreover, alternative polyadenylation plays an important role in neurodegenerative diseases. For example, in a recent transcriptome wide association study, 354 genes were found to be associated with alternative polyadenylation in 11 brain disorders including Parkinson’s disease and amyotrophic lateral sclerosis (ALS) (Cui et al., 2023).

## 5 Mislocalized RNA in neurodegenerative diseases

mRNAs spontaneously form secondary structures, such as stem loops, hairpins, helices, pseudoknots and unstructured regions. These structures, along with zipcodes, create novel binding interfaces for RBPs. For example, a 54-nucleotide zipcode within a secondary structure in the *beta-actin* mRNA pairs with an unfolded region to create a binding site for the Zipcode binding protein 1 (ZBP1) protein. The ZBP1 bound RNA is then directed to actin-rich protrusions in neurons and fibroblasts ((Huttelmaier et al., 2005) reviewed in (Fernandopulle, Lippincott-Schwartz and Ward, 2021)). Generally within neurons, mRNAs that localize to axons and dendrites are richer in secondary structure and have a longer half-life than their counterparts in the soma (Tushev et al., 2018). A common feature of neurodegenerative diseases is protein aggregation. Mislocalization of RBPs to protein aggregates may prevent accurate RNA transport and localized translation. For example, mislocalization of specific mRNAs leads to impaired local protein synthesis associated with the pathology of ALS and other motor neuron diseases (reviewed in (Blanco-Urrejola et al., 2021)). Moreover, deregulated localization of RNA in Alzheimer’s disease (AD), where the translation regulatory *BC200* RNA is clustered in AD brain, contributes to synaptic dysfunction and dendritic regression (Mus, Hof and Tiedge, 2007). Yet, we are just beginning to understand how RNAs are transported to their intracellular destination. The study of mislocalized RNAs in neurodegenerative diseases not only improves our understanding of these diseases, but also opens potential targets for therapeutic intervention.

## 6 Methods to assess RNA localization

As numerous as the RNA localization mechanisms are, so is the number of methods to determine and understand them. Firstly, RNA from different cellular fractions can be analyzed by RNA sequencing techniques (reviewed in (Stark, Grzelak and Hadfield, 2019)). The high sensitivity of this method enables the detection of a broad spectrum of RNA species. Microdissection techniques, especially in brain tissue, enable the isolation of specific neuronal compartments and can provide insights into localized RNA processing in neurons. However, it should be emphasized that a sufficient number of biological replicates is necessary to ensure the reliability of the data. Next, staining methods such as fluorescence *in situ* hybridization (FiSH) and single-molecule FiSH allow the direct visualization of RNA molecules in cells (reviewed in (Jensen, 2014)). These methods provide valuable spatial information about individual RNA transcripts, but generally do not have the capacity for high-throughput analysis. High-throughput techniques such as RNAi screens (reviewed in (Mohr and Perrimon, 2011)) and massive parallel reporter assays (reviewed in (Zheng and VanDusen, 2023)) are critical for understanding gene function and regulation on a large scale. Furthermore, there are also long-read sequencing methods. These offer better accuracy and sensitivity in the identification of RNA isoforms (Byrne et al., 2017). Despite their potential, these methods are not yet widely used due to scalability and cost issues indicating the need for further refinement and development of experimental tools. For example, one recent study used photocatalytic proximity labelling of stress granules to comprehensively investigate the stress granule-transcriptome in live mammalian cells (Ren, Tang, Peng and Zou, 2023). Additionally, bioinformatic tools for predicting RNA localization signals are important to support experimental designs but cannot completely replace experimental methods.

## 7 Discussion and conclusion

The dynamic world of RNA, from localization to function, is an emerging field in cell biology and disease pathology. Recently, sensitive techniques that even detect single RNA molecules have been developed to deepen our understanding of RNA regulation. In addition to the selection of the methods to be carried out, other aspects must be considered to understand the function of RNA and its localization. These aspects include RNA modification, which has a profound impact on RNA transport and localization (reviewed in (Qiu, Jing, Li and Han, 2023)). Besides the above mentioned zipcodes and alternative polyadenylation, other RNA modifications occur. For example, a recent study showed that NAD capping of RNAs, an addition of nicotinamide adenine dinucleotide to the 5′end of the RNA, can occur that affects the stability of the RNA (Jiao et al., 2017). Such modifications may affect the subcellular localization of RNA as well and should be studied in more detail. Ongoing research on cis-elements, RNA-protein interactions, and localization signals will further elucidate the effects of RNA localization on cell function and dysfunction, especially in neurodegenerative diseases. Therefore, methods to more precisely follow single RNAs in living cells need further improvement and development. Moreover, existing methods may be combined to provide deeper insights into the world of RNA dynamics and function.
